# Discovery of MicroRNAs Associated with Myogenesis by Deep Sequencing of Serial Developmental Skeletal Muscles in Pigs

**DOI:** 10.1371/journal.pone.0052123

**Published:** 2012-12-21

**Authors:** Xinhua Hou, Zhonglin Tang, Honglin Liu, Ning Wang, Huiming Ju, Kui Li

**Affiliations:** 1 Department of Animal Genetics, Breeding and Reproduction, College of Animal Science and Technology, Nanjing Agricultural University, Nanjing, P.R. China; 2 Key Laboratory of Farm Animal Genetic Resources and Germplasm Innovation of Ministry of Agriculture, Institute of Animal Science, Chinese Academy of Agricultural Sciences, Beijing, P.R. China; 3 College of Animal Science and Technology, Northeast Agricultural University, Haerbin, P.R. China; University of Massachusetts Medical, United States of America

## Abstract

MicroRNAs (miRNAs) are short, single-stranded non-coding RNAs that repress their target genes by binding their 3′ UTRs. These RNAs play critical roles in myogenesis. To gain knowledge about miRNAs involved in the regulation of myogenesis, porcine longissimus muscles were collected from 18 developmental stages (33-, 40-, 45-, 50-, 55-, 60-, 65-, 70-, 75-, 80-, 85-, 90-, 95-, 100- and 105-day post-gestation fetuses, 0 and 10-day postnatal piglets and adult pigs) to identify miRNAs using Solexa sequencing technology. We detected 197 known miRNAs and 78 novel miRNAs according to comparison with known miRNAs in the miRBase (release 17.0) database. Moreover, variations in sequence length and single nucleotide polymorphisms were also observed in 110 known miRNAs. Expression analysis of the 11 most abundant miRNAs were conducted using quantitative PCR (qPCR) in eleven tissues (longissimus muscles, leg muscles, heart, liver, spleen, lung, kidney, stomach, small intestine and colon), and the results revealed that ssc-miR-378, ssc-miR-1 and ssc-miR-206 were abundantly expressed in skeletal muscles. During skeletal muscle development, the expression level of ssc-miR-378 was low at 33 days post-coitus (dpc), increased at 65 and 90 dpc, peaked at postnatal day 0, and finally declined and maintained a comparatively stable level. This expression profile suggested that ssc-miR-378 was a new candidate miRNA for myogenesis and participated in skeletal muscle development in pigs. Target prediction and KEGG pathway analysis suggested that bone morphogenetic protein 2 (BMP2) and mitogen-activated protein kinase 1 (MAPK1), both of which were relevant to proliferation and differentiation, might be the potential targets of miR-378. Luciferase activities of report vectors containing the 3′UTR of porcine BMP2 or MAPK1 were downregulated by miR-378, which suggested that miR-378 probably regulated myogenesis though the regulation of these two genes.

## Introduction

Myogenesis is a complex process that includes proliferation, differentiation, and formation of myotubes and myofibers. In the developing vertebrate embryo, mononuclear proliferative myoblasts exit the cell cycle irreversibly and enter the differentiation phase. Several myoblasts subsequently fuse and form multinuclear myotubes. Finally, myofibers are formed following the distribution of nuclei to the edge of membrane. These molecular events are orchestrated by myogenic regulatory factors and miRNAs. The miRNAs that are expressed abundantly in skeletal muscle cells or myocardial cells are called myomiRs. MiR-1, miR-206 and miR-133 are classified as myomiRs, as they play important roles in the regulation of muscle development and differentiation. Previous experiments showed that miR-1 and miR-206 promoted the differentiation of myoblasts, while miR-133 promoted proliferation [1], [2]. Chromatin immunoprecipitation showed that the myogenic regulatory factors MyoD and myogenin upregulated the expression of miR-1-1, miR-1-2, miR-133a-1, miR-133a-2 and miR-206 in myotubes [3]. The myocyte enhancer factor-2 (MEF2) regulated the expression of myomiRs via a muscle-specific enhancer in an intron lying between the coding regions of miR-1-2 and miR-133a-1. There were also MEF2 and bHLH binding sites in a muscle-specific enhancer separating the miR-1-1 and miR-133a-2 coding regions [4]. Thus, the expression of myomiRs is regulated by myogenic factors, such as MyoD, and the myomiRs mediate muscle development or differentiation by regulating downstream target genes. Also, miRNAs have counter-effects on myogenic factors. Naguibneva et al. reported that miR-181 alleviated the repression of MyoD by downregulating Hox-A11, which represses the expression of MyoD, which, in turn, triggered the expression of muscle markers [5]. Pax3, which plays a role in a number of aspects of embryonic myogenesis, can be targeted by miR-27b, leading to its downregulation and early differentiation. Pax3 levels are maintained when miR-27b is inhibited, and inhibition of miR-27b also leads to more proliferation and delays the onset of differentiation [6]. It seems that miR-24 has a functional role in the regulation of differentiation, as it can be upregulated during the early stages of differentiation but is maintained in adult terminally differentiated cardiac and skeletal muscles. This miRNA is also upregulated during cardiac hypertrophy and induces cardiomyocyte hypertrophy [7]. MiRNAs can also control the fiber type of skeletal muscles. Mice that have a double deletion of miR-208b and miR-499 show a substantial loss of slow myofibers, while overexpression of miR-499 under control of the MCK promoter converts all of the fast fibers to the slow phenotype in the soleus muscles, resulting in an enhanced endurance phenotype, with miR-499 transgenic mice running more than 50% longer than their wild-type littermates [8]. Many other miRNAs also take part in the regulation of muscle development. MiR-221 and miR-222 are downregulated during the transition from proliferation to differentiation of both primary and established myogenic cells. The cell cycle inhibitor p27 can be targeted by both miR-221 and miR-222, and overexpression of these miRNAs delays withdrawal from the cell cycle and differentiation, due to a reduction in sarcomeric proteins [9]. MiR-155 can regulate the expression of Olfactomedin-like 3 (OLFML3), which may affect porcine prenatal skeletal muscle development [10]. Although an increasing body of evidence shows that miRNAs play important roles in the regulation of skeletal muscle development, precise regulatory mechanisms of the biological functions of most miRNAs remain unclear, and most research is restricted to myomiRs, such as miR-1, miR-133 and miR-206.

Developing pigs have a much longer embryonic period than mice; therefore, their development can be divided into more stages to obtain a more precise understanding of embryonic myogenesis. Many research groups have identified highly abundant and novel miRNAs from porcine skeletal muscles by cloning, microarray assays or deep sequencing methods [11], [12], [13]. However, all of these studies chose notably limited growth periods for analysis. As the development of skeletal muscles is so complicated, with many different genes taking part in the regulation of proliferation and differentiation during the different growth periods, collection of samples in only a few growth periods may fail to detect many miRNAs that are involved in muscle development. To investigate myomiRs comprehensively, we collected skeletal muscle at 18 developmental stages (33-, 40-, 45-, 50-, 55-, 60-, 65-, 70-, 75-, 80-, 85-, 90-, 95-, 100-, and 105-day post-gestation, 0- and 10-day postnatal piglets, and adult pigs) and pooled all RNAs to identify known and novel miRNAs involved in myogenesis using the Solexa sequencing method. The spatial expression patterns of the 11 most abundant miRNAs were analyzed in eleven adult tissues by qPCR. The results showed that ssc-miR-378 was abundantly expressed in skeletal muscles. We detected the dynamic expression of ssc-miR-378 in prenatal and postnatal skeletal muscle. The targets of miRNA-378 were predicted, and the predicted targets were analyzed to investigate their involvement in known pathways. The results of luciferase activities suggested that miR-378 could target the wild type 3′UTRs of porcine BMP2 or MAPK1, but not the mutation ones in which the binging sites were deleted.

## Results

### An Overview of the Small RNAs (sRNAs) Library

**Figure 1:**
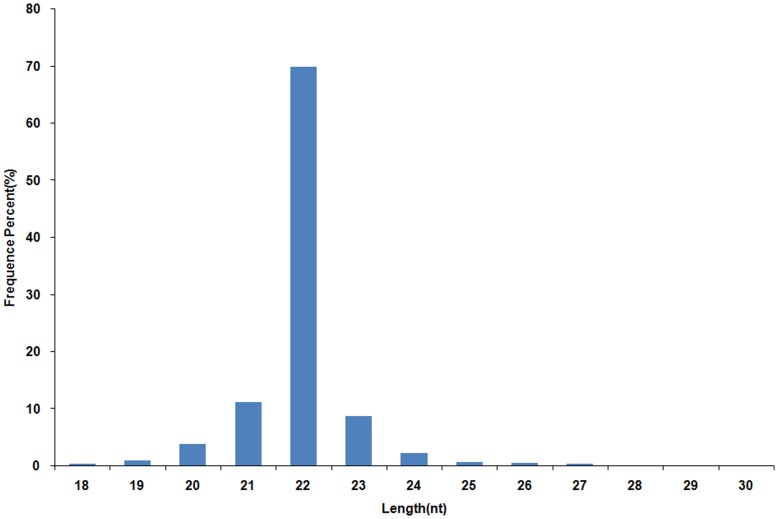
Sequence length distribution.

Solexa sequencing was conducted to determine the abundance of miRNAs in porcine skeletal muscles using pooled RNAs. A total of 23,813,176 reads were obtained of which 19,937,559 were high-quality. After eliminating reads without a 3′ primer (14,444) or insert tag (8,776), reads with polyA (77) or 5′ primer contaminants (1,528) and reads shorter than 18 nt (57,754), 19,854,980 clean reads of total sRNAs representing 415,165 unique sRNAs remained. These reads accounted for 99.59% of the high-quality reads. Also, sRNAs sequences ranged from 21∼23 nt in length with a distribution peak at 22 nt, which was consistent with the common size of miRNAs (Figure 1). After further removal of sRNA reads that matched to rRNA, snoRNA, piRNA and tRNA (see detailed information in Table S1) in the Rfam (9.1) and NCBI GenBank databases, there were 14,034,350 total reads and 3407 unique miRNAs representing 172 precursor and 197 mature miRNAs.

### Sequence Variants in Mature miRNAs

Our Solexa results showed that many miRNAs possessed variations in sequence length and single nucleotide polymorphisms (SNPs) that might be attributed to post-transcriptional modifications of the RNA. These miRNA variations were referred to as isomers, and all of the 110 known miRNAs had isomers (Table S2–1). Position 2∼8 of mature miRNAs is known as the seed region, which is highly conserved; alterations in the nucleotides in this region may result in alterations in the recognition between miRNAs and their target genes. In our results, a total of 58 mature miRNAs displayed SNPs in the seed region (Table S2–2), and high-abundance miRNAs had more variants than did low-abundance miRNAs. Thus, the results suggest that high-abundance miRNAs may perform more diverse functions in skeletal muscles. Moreover, a large number of variants may increase the number of target genes for each miRNAs. As we performed Solexa sequencing using pooled RNA, it is possible that variants for a certain miRNA might be derived from different growth periods. It could also be speculated that certain variants might play essential roles in a certain period. More investigations should be performed to determine whether miRNA variants are specific to particular developmental stages and, if so, to investigate the biological functions of these variants.

### Prediction of Novel miRNAs

BLASTN searches against miRBase (17.0) revealed that 78 sequences that were considered as predicted novel miRNAs from our Solexa results had no similarity to known porcine miRNAs. All of the novel miRNAs ranged from 21∼24 nt in length, while 22-nt sequences accounted for most sequences (51.28%) followed by 23 nt (26.92%), 21 nt (17.95%) and 24 nt (3.85%). Copy numbers of novel miRNAs were at a relatively low level: 68 putative novel miRNAs were sequenced below 100 times, 9 were below 1000 times, and only 1 was above 1000 times. An important feature of miRNAs is that their precursor sequences can be folded into secondary hairpin structures. Potential novel mature miRNAs with their flanking regions were folded using Mfold (version 3.5), and the folding free energy (ΔG) ranged from −17.10 to −62.20 kcal/mol. The identified novel miRNAs that could not form typical secondary structures were discarded, and the remaining miRNAs were shown in Table S3. Some mature miRNAs possessed more than one predicted hairpin structure (such as ssc-miR-new_08 and ssc-miR-new_21). Among the potential novel miRNAs, 12 miRNAs perfectly matched known mature miRNAs in miRbase (Table 1); however, only 6 of these miRNAs’ precursors matched corresponding known precursors and were considered as the same miRNAs in pig. These 6 miRNAs were ssc-miR-new_03, ssc-miR-new_25, ssc-miR-new_26, ssc-miR-new_45, ssc-miR-new_74, and ssc-miR-new_77, and their corresponding known miRNAs were hsa-miR-3613-5p, bUa-miR-2366, hsa-miR-3591-5p, bUa-miR-2411*, bUa-miR-2483, and age-miR-508. Precursors of the other 6 matched miRNAs (ssc-miR-new_06, ssc-miR-new_18, ssc-miR-new_27, ssc-miR-new_28, ssc-miR-new_53 and ssc-miR-new_75) were not similar to any precursors in miRbase. It was interesting that ssc-miR-new_06, ssc-miR-new_18, ssc-miR-new_27 and ssc-miR-new_53 matched the same mature miRNA, hsa-miR-574-5p, but their precursors had no sequence homologous to each other or to precursors of has-miR-574-5p. The same situation, which was mentioned above, existed for ssc-miR-new_28 and ssc-miR-new_75 comparing to ssc-miR-378. This kind of miRNA might represent different members from the same miRNA family. The remaining 66 putative novel miRNAs had no remarkable similarity to any known miRNAs in miRbase, and the copy numbers were low. It was probable that all of these unmatched miRNAs were pig-specific miRNAs.

**Table 1:** Novel miRNAs matched to known miRNAs.

Category	Mature sequence	Length	Known miRNA	Mature sequence	Length
ssc-miR-new_03	UGUUGUACUUUUUUUUUUGUUC	22	hsa-miR-3613-5p	UGUUGUACUUUUUUUUUUGUUC	22
ssc-miR-new_06	UGAGUGUGUGUGUGUGAGUGUA	22	hsa-miR-574-5p	UGAGUGUGUGUGUGUGAGUGUGU	23
ssc-miR-new_18	UGAGUGUGUGUGUGUGAGUGUAG	23	hsa-miR-574-5p	UGAGUGUGUGUGUGUGAGUGUGU	23
ssc-miR-new_25	UGGGUCACAGAAGAGGGUCUGG	22	bUa-miR-2366	UGGGUCACAGAAGAGGGUCUGG	22
ssc-miR-new_26	UUUAGUGUGAUAAUGGCGUUUG	22	hsa-miR-3591-5p	UUUAGUGUGAUAAUGGCGUUUGA	23
ssc-miR-new_27	AGAGUGUGUGUGUGUGAGUGUGU	23	hsa-miR-574-5p	UGAGUGUGUGUGUGUGAGUGUGU	23
ssc-miR-new_28	ACUGGACUUGGAGUCAGAAGU	21	ssc-miR-378	ACUGGACUUGGAGUCAGAAGGC	22
ssc-miR-new_45	UGAACUGUCAUACUCCCACAUCC	23	bUa-miR-2411*	UGGAGUGACUGUCAGAUGCAGCCA	24
ssc-miR-new_53	UGAGUGUGUGUGUGUGAGUGUGA	23	hsa-miR-574-5p	UGAGUGUGUGUGUGUGAGUGUGU	23
ssc-miR-new_74	AAACAUCUGGUUGGUUGAGAGA	22	bUa-miR-2483	AAACAUCUGGUUGGUUGAGAGA	22
ssc-miR-new_75	CUGGACUUGAAGUCAGAAGGC	21	ssc-miR-378	ACUGGACUUGGAGUCAGAAGGC	22
ssc-miR-new_77	ACUGUCACCUUUUUGAGUAGA	21	age-miR-508	CGAUUGUCACCUUUUUGAGUAGA	23

### Tissue Distribution of the Top 11 Most Abundantly Expressed miRNAs in the sRNA Library

**Figure 2:**
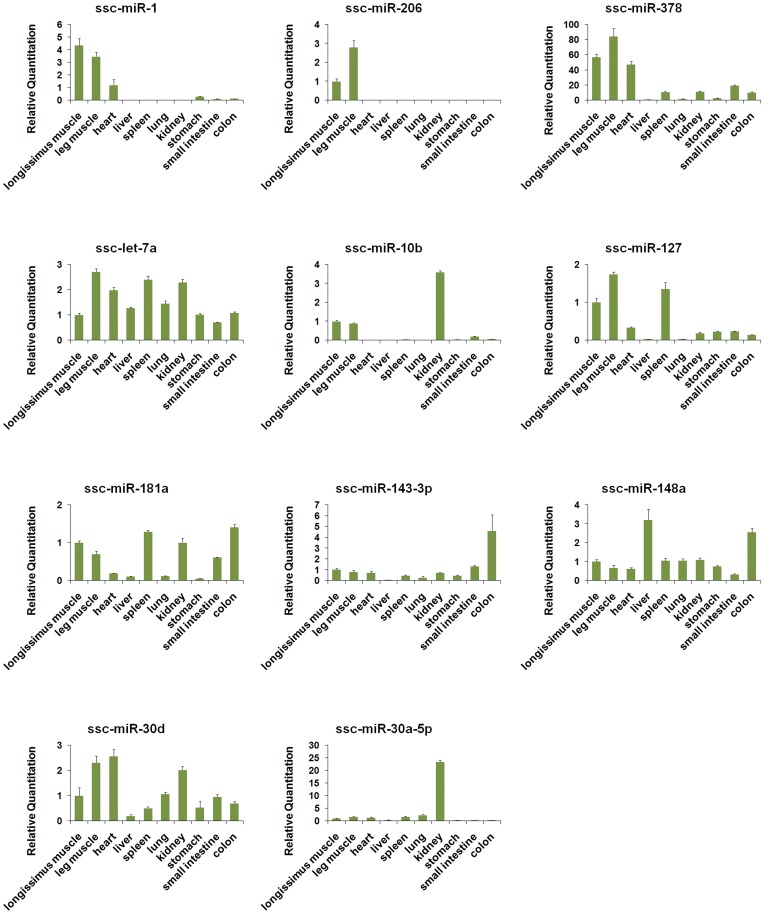
Expression profiles of several high-abundance miRNAs using stem-loop RT-PCR. Total RNAs were isolated from longissimus muscles, leg muscles, heart, liver, spleen, lung, kidney, stomach, small intestine and colon. The expression levels were analyzed using the ΔΔ Ct method and normalized against U6. Each sample was run in triplicate, and the calculation standard deviation was shown.

The read numbers from deep sequencing are often regarded as a reliable quantification of miRNA expression, and read numbers of miRNAs in our Solexa anlysis were shown in Table S4. Highly abundant miRNAs might play more important roles in regulation of skeletal muscles development or maintenance of vital physiological functions. As our Solexa sequencing results only revealed the abundance of miRNAs in longissimus muscles, we subsequently used stem-loop qRT-PCR to measure the expression pattern of highly abundance miRNAs in longissimus muscles, leg muscles, heart, liver, spleen, lung, kidney, stomach, small intestine and colon (Figure 2). Ssc-let-7a was highly expressed in all the different organs, which suggested that this type of miRNA might participate widely in regulations throughout the whole body. Ssc-miR-206 was expressed only in skeletal muscles, while ssc-miR-1 was expressed only in the skeletal muscles and heart. These two miRNAs were considered as myomiRs. Although ssc-miR-378 was expressed in almost all the organs, it was much more highly expressed in skeletal muscles and heart than in other organs. We speculated that ssc-miR-378 might be a vital miRNA in skeletal muscles. Other miRNAs, such as ssc-miR-10b, ssc-miR-181a, ssc-miR-30d and ssc-miR-127, were also expressed at a high level in skeletal muscles, but they were not myomiRs. Ssc-miR-10b was expressed in the kidney; ssc-miR-181a was expressed in the spleen, kidney, small intestine and colon; and ssc-miR-127 was also expressed in the spleen. Several miRNAs expressed at a relatively low level in skeletal muscles included ssc-miR-143-3p, expressed mainly in the colon; ssc-miR-148a, expressed in the liver and colon; and ssc-miR-30a-5p, expressed at extremely high levels in the kidney.

### Dynamic Expression of Ssc-miR-378 During Various Ontogenetic Development

**Figure 3:**
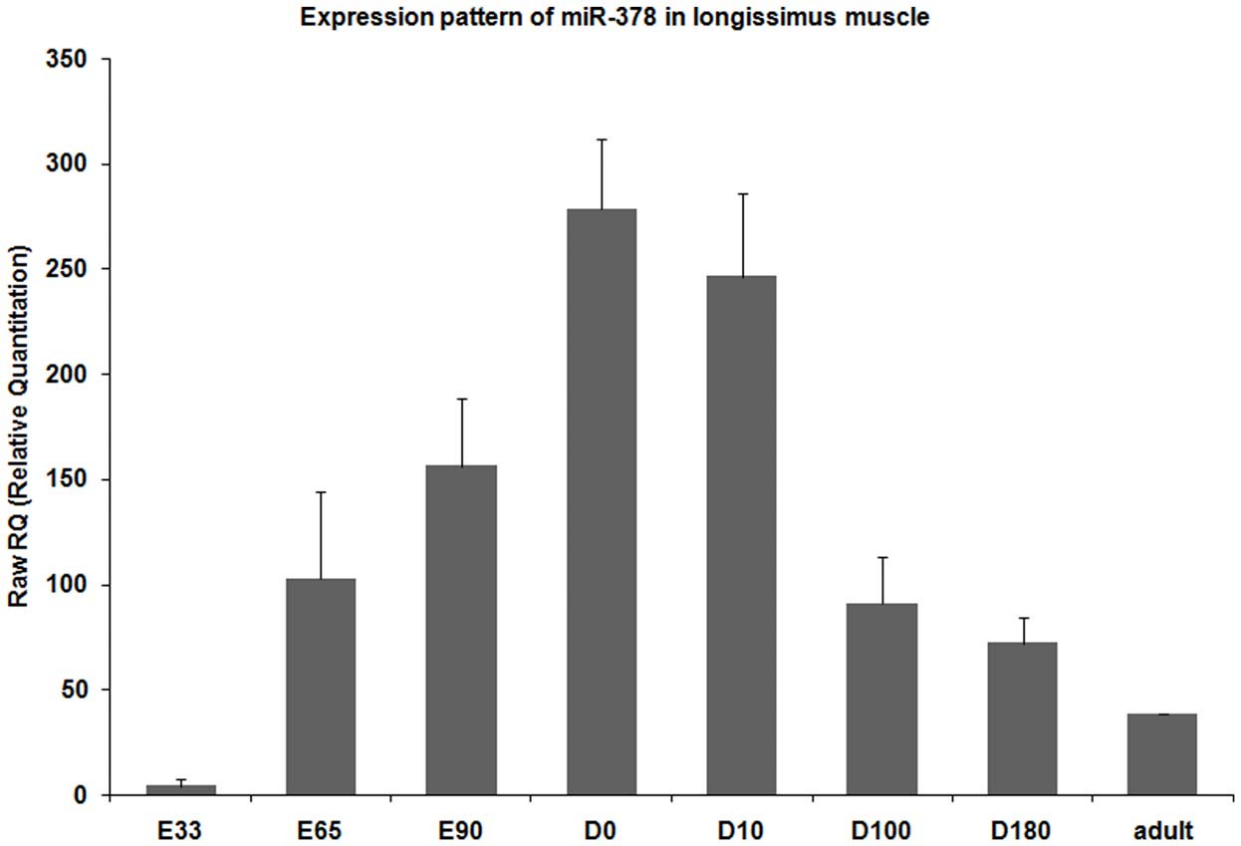
Expression profiles of miR-378 during various development periods. Total RNAs was isolated from porcine longissimus muscle during different dpc (E) and postnatal days (D), and qPCR was performed using stem-loop RT-PCR. The expression levels were analyzed using the ΔΔ Ct method and normalized against U6. Each sample was run in triplicate, and the calculation standard deviation was shown.

To gain further understanding of the biological role of ssc-miR-378 in the process of myogenesis, total RNAs from longissimus muscles of adult pig, fetuses in the 33, 65, 90 days post-coitus (dpc) stages and piglets in the 0, 10, 100, and 180 postnatal days were isolated, and qPCR was performed to identify its expression pattern. As shown in Figure 3, the expression of ssc-miR-378 was extremely low in 33 dpc, increased in 65 and 90 dpc, reached its maximum level at postnatal day 0, decreased during postnatal individual growth and was maintained at a stable level in the adult stage. The high expression periods of ssc-miR-378 were consistent with that of myofiber formation, suggesting that it might be an important miRNA in promoting muscle fiber formation.

### Target Prediction of miR-378 and GO Analysis

To acquire more knowledge concerning the biological role of miR-378 in skeletal muscle differentiation, regulatory targets were predicted using TargetScan. As porcine genes were not included in the current versions of TargetScan, prediction was performed using human miRNA:mRNA 3′UTR interaction. A total of 191 predicted target genes were obtained and the DAVID bioinformatics resource (version 6.7) was used to identify pathways that may be involved in myogenesis. Finally, the BMP2 and MAPK1 attracted us mostly. MAPK1, which was also named as extracellular signal-regulated kinase 2 (ERK2), participated in the MAPK pathway, which was involving in the regulation of proliferation. In this pathway, insulin-like growthfactor 1 (IGF1) binds the insulin-like growthfactor 1 receptor (IGF1-R), which results in phosphorylation of the receptor by activating the tyrosine kinase domains within the β-subunit. SH2-containing proteins (Shc) becomes phosphorylated on tyrosine residues by the activated IGF1-R and subsequently acts as a docking site for SH2 domain of growth factor receptor-bound protein 2 (GRB2). Son of sevenless (SOS) binds to the SH3 domain of GRB2 and forms the GRB2-SOS complex [14]. Phosphorylated Shc, when combined with the GRB2-SOS complex, activates the Ras/Raf/MAPK pathway, which leads to mitogenesis [15]. It was possible that miR-378 repressed proliferation of myoblasts by inhibiting this pathway and thus promoted differentiation in an indirect manner. Bone morphogenetic proteins (BMPs) are multifunctional growth factors within the transforming growth factor β (TGF-β) super family that were identified based on their ability to initiate ectopic bone formation in adult animals [16]. BMP2 inhibits terminal differentiation of C2C12 myoblasts and converts them into osteoblast lineage cells [17], [18], [19]. Therefore, we concerned about these two genes and verified whether they were the candidate targets of miR-378.

### BMP2 and MAPK1 are Endogenous Target of miR-378

**Figure 4:**
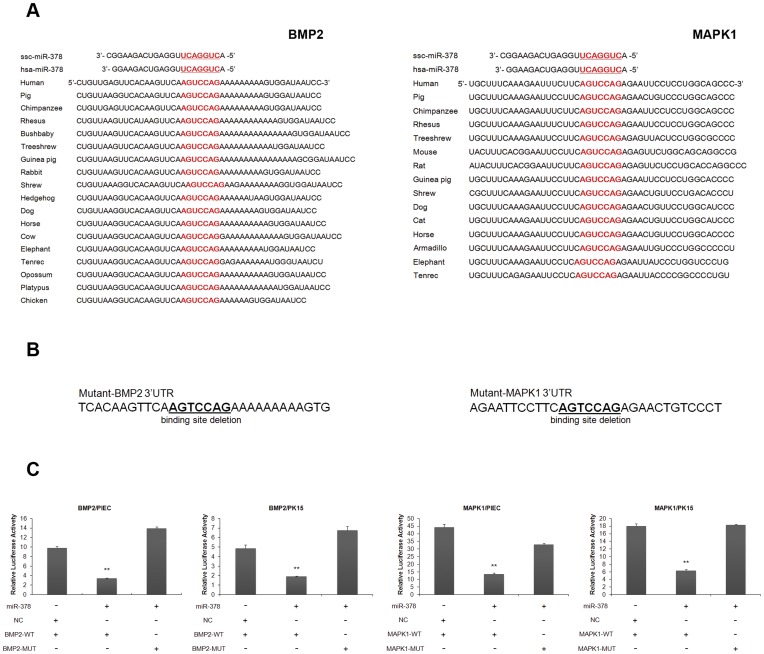
BMP2 and MAPK1 are potential target of miR-378. (A) Predicted miR-378 binding site (highlighted in red) in the 3′UTR of BMP2 or MAPK1 showing species conservation. (B) Schematic of the predicted miR-378 binding sites (underlined) in the 3' UTR of BMP2 or MAPK1. The region of binding sites were deleted in the mutant 3' UTR reporters. (C) Luciferase activety assay of the wild-type (WT) or mutant (MUT) 3′UTR of BMP2 and MAPK1 using a dual luciferase reporter system in PIEC or PK15 cell lines following co-transfection with miR-378 mimics or negative control mimics (NC). Data is derived from triplicate tranfectants of 3 independent experiments (**p<0.01).

**Figure 5:**
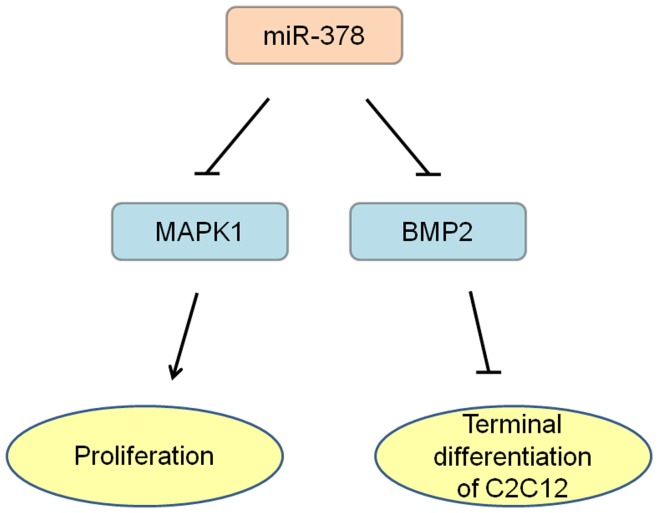
Presumed regulatory network of miR-378 in myogenesis.

As binding site region plays an important role in recognition between target genes and miRNAs, we compared binding site region along with the flanking sequence within the 3′UTR of both BMP2 and MAPK1 across various vertebrate species, and found that all the binding sites were highly conserved (Figure 4A). To determine the regulation role of miR-378 against BMP2 and MAPK1, luciferase reporter vectors containing wild-type or mutant 3′UTR of BMP2 or MAPK1 (Figure 4B) were constructed and co-transfected with miR-378 mimics or negative control mimics into PIEC and PK15 cell lineages. As shown in Figure 4C, luciferase activities of both wild-type BMP2 and MAPK1 reporters co-transfected with miR-378 mimics were reduced significantly compared to that with negative control mimics or mutant reporters with miR-378 mimics. These results suggested that miR-378 directly targeted the BMP2 and MAPK1 3′UTR, and might regulate myogenesis in the manner of acceleration of the differentiation and inhibition of proliferation (Figure 5).

## Discussion

Solexa sequencing was performed to identify miRNAs in porcine skeletal muscles. A total of 197 known and 78 potential novel miRNAs were identified. Next, we analyzed isomiRs of known miRNAs in our results, as isomiRs might have functions in animals [20]. Nearly 27.7% miRNAs had isomiRs, and only 0.14% were in the seed region, showing that low mutant frequency and high conservation existed in this region. There were always 1∼2 nucleotide differences in length at the 5′ or 3′ terminal of isomiRs for the same miRNA, which might be attributable to variable cleavage of pri- or pre-miRNA by Drosha or Dicer. Potential novel miRNAs were identified by genome location and secondary structure predictions. The number of reads for these miRNAs were usually indicative of low abundance, which might suggest that these low-abundance novel miRNAs had little effect on skeletal muscle myogenesis. Nielsen et al. [21] showed that ssc-miR-1 and ssc-miR-206 are expressed at extremely high levels in the longissimus muscles of Danish Landrace/Yorkshire crossbred pigs of age 1.5–2 years; however, the abundance of other miRNAs was relatively low. In our results, the abundance of ssc-miR-1 and ssc-miR-206 was extremely high, which was consistent with their results. However, there also existed many miRNAs for which the expression levels were different between the two studies. An example was ssc-miR-378, which we found at a much higher abundance than their study found. In addition, we found that ssc-miR-148a was expressed highly in the longissimus muscles, while it was expressed in an extremely low level in their results. These differentially expressed miRNAs may be highly expressed during fetal stages and play vital roles in the regulation of fetal myogenesis. These results also demonstrate that including many developmental stages in our analysis was beneficial to discovering the range of differentially expressed miRNAs among serial development periods.

Ssc-miR-206, ssc-miR-1 and ssc-miR-378 had the most reads in our Solexa results. Currently, miR-1, miR-133 and miR-206 are considered as myomiRs. MiR-1 and miR-133 are expressed both in cardiac and skeletal muscles [22], whereas miR-206 is expressed only in skeletal muscles [23], [24]. The qPCR results of ssc-miR-1 and ssc-miR-206 were consistent with previous reports. Although ssc-miR-133, another type of myomiR, could also be detected in our Solexa results, the reads were moderate and were not as abundant as that of ssc-miR-1 and ssc-miR-206. This finding may suggest that differentiation might be predominant compared to proliferation during the period from 33 dpc to postnatal 10 day, as miR-133 mainly plays a role in the regulation of muscle proliferation [1]. Lee et al. proved that miR-378 enhanced cell survival, tumor growth, and angiogenesis by repressing the expression of two tumor suppressors, Sufu and Fus-1 [25]. MiR-378 can also mediate the differentiation of MC3T3-E1, which is a type of osteoblastic cell, through modulating nephronectin, which will promote osteoblast differentiation and bone nodule formation when overexpressed [26]. These lines of evidence suggest that miR-378 plays an important role in regulating cellular proliferation and differentiation. Our qPCR results showed that ssc-miR-378 was expressed highly in cardiac and skeletal muscles, and it may exert important physiological effects on muscle development. During the development of skeletal muscles in the fetal period, primary fibers formed first; later, secondary fibers were generated deriving from myoblasts around the primary fibers. Nearly 95% of final muscles fibers arose from secondary fibers, and the diameter of primary fibers determined the amount of attached secondary fibers. Therefore, both fibers can influence the total number of final matured muscles fibers. Porcine primary fibers mainly formed at 38 to 64 dpc and secondary fibers at 54 to 90 dpc [27]. Previous research showed that 33, 65 and 90 days’ gestation can be considered as critical time points in prenatal skeletal muscle development [28]; therefore, we selected those three stages to analyze the expression pattern of ssc-miR-378 during fetal periods. The qPCR showed that ssc-miR-378 was expressed at low levels at 33dpc and high at 65 and 90 dpc, which was consistent with the pattern of fiber formation during fetal development, suggesting that this kind of miRNA might be involved in muscle fiber formation. Recently, a report showed that MyoD could promote expression of miR-378, which suppressed MyoR, a MyoD inhibitor, by recognizing its 3′ UTR, and differentiation was accelerated because of relief of MyoD inhibition [29]. This finding also confirmed the biological function of miR-378 in myoblast differentiation. Other reports suggested that there exist tertiary fibers, which were distinct from primary and secondary fibers for their myosin composition, during the early post-natal period, resulting in a third wave of fiber increase [30], [31]. Maintenance of a relatively high level of expression of ssc-miR-378 during postnatal days 0 to 10 might be closely related to this tertiary fiber wave. In summary, ssc-miR-378 might play an important role in the regulation of myogenesis, especially in fiber formation in both the fetal and newborn periods.

Moreover, other miRNAs were also expressed abundantly in skeletal muscles, including ssc-let-7a, ssc-let-7c, ssc-let-7f, ssc-miR-143-3p, ssc-miR-10b, ssc-miR-148a, ssc-miR-127, ssc-miR-30d, ssc-miR-30a-5p, and ssc-miR-181a. The let-7 family is a conserved family of miRNAs, the members of which behave as tumor suppressors [32]. Let-7a inhibits cell growth and tumor formation [33], while let-7f inhibits the invasive and metastatic potency of gastric cancer lines by downregulation of MYH9 (a metastasis-associated gene) [34]. In prostate cancer, miR-143 decreased proliferation and increased apoptosis [35]. The oncogene KRAS can promote proliferation and inhibit apoptosis through activation of the RAS/RAF/MEK/ERK signaling pathway. Suppression of KRAS by miR-143 leads to inhibition of ERK 1/2 phosphorylation and, consequently, the reduction of cell numbers [36]. MiR-10b has a positive effect on migration and invasion in ESCC (esophageal squamous cell carcinoma) and breast cancer cells [37], [38]. MiR-148a inhibits cell growth and attenuates migration and invasion in prostate cancer PC3 cells [39]. Zhang et al. revealed that miR-148a positively regulated myogenic differentiation by downregulation of ROCK1 recently [40]. MiR-127 is silenced in cancer cells but normally expressed in fibroblasts, and it can suppress the expression of proto-oncogene BCL6, which endows miR-127 with the characteristic of a tumor suppressor [41]. The oncogene B-Myb is suppressed by miR-30, leading to induction of RB-dependent senescence [42]. MiR-181a is involved in the establishment of skeletal muscle differentiation [5] and repression of tumor growth [43]. None of the miRNAs mentioned above are myomiRs, but they might play important roles in the regulation of myogenesis because of their relationship with proliferation, differentiation, and apoptosis of cells or tumors.

Finally, target genes of miR-378 were predicted, and KEGG pathway analysis suggests that this miRNA is involved in the regulation of the MAPK signaling pathway. C2C12 myoblasts proliferate in high-serum concentrations media and differentiate when transferred to low-serum media. MyoD, an important regulator of myoblast differentiation, is expressed in low- and high-serum media, whereas it is inactive in high-serum media because of its interaction with an excess of CD1-cdk4, which is positively regulated by the MEK- ERK 1, 2 kinase pathway. Differentiation is triggered in the low-serum condition as CD1 amounts decrease and MyoD becomes activated [44], [45], [46]. Therefore, miR-378 probably controls myoblast differentiation by regulation of this pathway. BMP2 plays widely roles in regulation of migration and invasion of cancer cells or osteoblast differentiation [47], [48], [49]. BMP2/4 and their receptors are expressed in the myoblasts and myotubes of mouse embryonic tongues which are differentiating and maturating, implying that BMPs might have function in myoblast differentiation [50]. Meanwhile, reduction of BMP2 by specific siRNA could stimulate the differentiation of myoblasts in cultured tongues [51]. Previous research suggested that BMP2 not only converts the differentiation pathway into that of osteoblasts but also inhibits myogenic differentiation [17]. BMP2 inhibited the transcriptional activation of MyoD, myogenin, and terminal differentiation-associated genes including MCK [52]. Inhibitor of DNA binding/differentiation 1 (Id1) was identified as a gene for inhibition of myogenesis and as a typical early response gene in BMP treatment in various types of cells in mice and humans. There was a 29-bp GC-rich element as a BMP2- responsive element in the 5-flanking region of the human Id1 gene and the expression of Id1 was stimulated within 1 h after the addition of BMP2 in C2C12 cells [19]. Thus, miR-378 may promote myoblasts differentiation by suppressing BMP2. MicroRNAs may regulate multi-targets simultaneously, which was in accordance with our result that miR-378 could downregulate both BMP2 and MAPK1 by recognizing their 3′UTR. We can also speculate that there still exist numerous targets interacting with miR-378 in the regulation of myogenesis.

## Materials and Methods

### Sample Collection and Isolation of RNA

The TongCheng adult pigs, piglets and pregnant sows during the specified days post-coitus were sacrificed at a local slaughterhouse according to the protocols set forth by HuBei Province, PR China for the Biological Studies Animal Care and Use Committee. The sacrificed piglets were from the postnatal days 0, 10, 100, and 180, and the fetuses from the pregnant sows were in the 33, 40, 45, 50, 55, 60, 65, 70, 75, 80, 85, 90, 95, 100, and 105 dpc. The longissimus muscles, colon, lung, liver, spleen, kidney, leg muscles, stomach, small intestine, and heart were dissected and snap-frozen in liquid nitrogen. Total RNAs were isolated from each sample using Trizol (Invitrogen) according to the manufacturer’s protocol.

### Solexa Sequencing and Data Analysis

Total RNAs were pooled from the longissimus muscles of fetuses (33, 40, 45, 50, 55, 60, 65, 70, 75, 80, 85, 90, 95, 100, and 105 dpc), piglets (postnatal days 0 and 10) and adult pigs. RNA integrity was measured on an Agilent 2100 Bioanalyzer system (Agilent). 18∼30 nt RNA fragments were excised and purified from a PAGE gel, and adaptors were ligated to their 5′ and 3′ ends using T4 RNA ligase. RT-PCR was performed using adaptor primers for 17 cycles and the 90 bp DNA fragments were subsequently isolated from agarose gel. Subsequently, the purified DNA was used directly for sequencing analysis using the Illumina Genome Analyzer (Illumina) according to the manufacturer's instructions. The 35-nt sequence tags were obtained from Solexa sequencing. After eliminating adaptor sequences, low-quality tags, sequences smaller than 18 bp and reads with no insertion, all of the clean tags were annotated and classified by comparing with the non-coding RNAs (rRNA, tRNA, scRNA, snRNA, snoRNA) in the GenBank (http://www.ncbi.nlm.nih.gov/) and Rfam (9.1) (http://rfam.sanger.ac.uk/) databases. Known miRNAs were identified by comparing our clean tags to mature miRNAs in miRBase (17.0) (http://www.mirbase.org/). The remaining non-annotated sRNA sequences were aligned against the porcine genome, and genomic sequences containing the sRNA with 80∼100 nucleotides of flanking sequences were used to predict hairpin structures using the Mfold program (http://mfold.rna.albany.edu). Only the sequences that could be folded into typical hairpin structures and were located in intergenic regions or introns were considered to be miRNA precursor loci of potential novel miRNAs in the porcine genome. All the sequence data have been submitted to the NCBI Sequence Read Archive (http://www.ncbi.nlm.nih.gov/Traces/sra/) under accession No.SRA052085.

### Quantitative PCR

The cDNA was synthesized using the RevertAid ™ First Strand cDNA Synthesis Kit (Fermentas) with the reverse transcription primers shown in Table S5, similar to a previously described protocol [53]. Each real-time PCR contained 2×SYBR® Premix Ex Taq ™ (TaKaRa), 50×ROX Reference Dye II, 0.2 µM forward and reverse primers, 2 µL template cDNA, and dH_2_O up to the final volume of 20 µL. Reactions were performed on a 7500 FAST Real-Time PCR System (Applied Biosystems). The qPCR primers for miRNAs and U6 were also shown in Table S5. Cycling conditions consisted of an initial, single cycle of 30s at 95°C followed by 40 cycles of 5s at 95°C and 34s at 60°C. All the data were analyzed by the 2^−ΔΔCT^ method using 7500 System SDS Software V 1.4.0.

### Target Prediction and KEGG Pathway Analysis

To determine the biological function of miR-378 in the regulation of myogenesis, TargetScan, an online program, was used for prediction of potential target mRNAs (http://www.targetscan.org/). The predicted genes were subsequently classified according to KEGG functional annotations using the DAVID Bioinformatics Resources (http://david.abcc.ncifcrf.gov/tools.jsp), and those potential target genes participating in the pathways related to cell proliferation and differentiation were selected and further analyzed.

### Target Vector Construction

The 3′UTR of BMP2 or MAPK1 containing miR-378 binding site was amplified from porcine genomic DNA by the method of PCR. All the primers were shown in the Table S6. Both PCR products were cloned into psiCHECK-2 (Promega) using the NotI and XhoI restriction sites. Mutant target vectors, which had a 7 base pair deletion in the binding sites, were obtained from Taihe Biotechnology (Beijing).

### Cell Culture and Dual Luciferase Reporter Assay

PIEC and PK15 were culture in DMEM complete medium (Gibco) supplemented with 10% FBS (Hyclone) and 1% penicillin/streptomycin (Gibco). The 60 nM miR-378 mimics/negative control duplexes (GenePharma) were co-transfected with 200 ng luciferase reporter containing BMP2/MAPK1 3′UTR using Lipofectamine 2000 reagent (Invitrogen) in 24-well plates. After transfection for 48 hours, all the cells were harvest and the luciferase ratio of Renilla/firefly was obtained using Dual Luciferase Assay System (Promega).

## Supporting Information

Table S1 **Information about small RNAs and the distribution of sequences.**
(XLS)

Table S2 **Detail information of base edit in known miRNAs.**
(XLS)

Table S3 **Predicted chromosomal positions and hairpin structures of novel miRNAs.**
(XLS)

Table S4 **Rank of known mRNAs in solexa sequencing.**
(XLS)

Table S5 **Primers for miRNA RT-qPCR.**
(DOC)

Table S6 **Primers for luciferase reporter construction.**
(DOC)
