# Supplementary material for: Discovery of MicroRNAs Associated with Myogenesis by Deep Sequencing of Serial Developmental Skeletal Muscles in Pigs
Source: PLoS One. 2012 Dec 21;7(12):e52123. doi: 10.1371/journal.pone.0052123 (PMC3528764; doi:10.1371/journal.pone.0052123)
Supplement: Table S5 — Primers for miRNA RT-qPCR. (DOC) [file pone.0052123.s005.doc]

**Table S5 Primers for miRNA RT-qPCR**

| miRNA ID | primer | sequence (5'→3') |
| --- | --- | --- |
|  |  |  |
| ssc-let-7a | SP | GGGTGAGGTAGTAGGTT |
|  | RT loop | CTCAACTGGTGTCGTGGAGTCGGCAATTCAGTTGAGAACTATAC |
| ssc-miR-10b | SP | GGGTACCCTGTAGAACC |
|  | RT loop | CTCAACTGGTGTCGTGGAGTCGGCAATTCAGTTGAGACAAATTC |
| ssc-miR-127 | SP | GGGTCGGATCCGTCTG |
|  | RT loop | CTCAACTGGTGTCGTGGAGTCGGCAATTCAGTTGAGGCCAAGCT |
| ssc-miR-181a | SP | GGGAACATTCAACGCTGT |
|  | RT loop | CTCAACTGGTGTCGTGGAGTCGGCAATTCAGTTGAGACTCACCG |
| ssc-miR-143-3p | SP | GGGTGAGATGAAGCA |
|  | RT loop | CTCAACTGGTGTCGTGGAGTCGGCAATTCAGTTGAGAGCTACAG |
| ssc-miR-148a | SP | GGGTCAGTGCACTACAG |
|  | RT loop | CTCAACTGGTGTCGTGGAGTCGGCAATTCAGTTGAGACAAAGTT |
| ssc-miR-30d | SP | GGGTGTAAACATCCCCGA |
|  | RT loop | CTCAACTGGTGTCGTGGAGTCGGCAATTCAGTTGAGGCTTCCAG |
| ssc-miR-30a-5p | SP | GGGTGTAAACATCCTCGA |
|  | RT loop | CTCAACTGGTGTCGTGGAGTCGGCAATTCAGTTGAGGCTTCCAG |
| ssc-miR-1 | SP | GGGTAGCTTATCAGACT |
|  | RT loop | CTCAACTGGTGTCGTGGAGTCGGCAATTCAGTTGAGTCAACATC |
| ssc-miR-206 | SP | GGGTGGAATGTAAGGAA |
|  | RT loop | CTCAACTGGTGTCGTGGAGTCGGCAATTCAGTTGAGTCACACAC |
| ssc-miR-378 | SP | GGGACTGGACTTGGAGT |
|  | RT loop | CTCAACTGGTGTCGTGGAGTCGGCAATTCAGTTGAGGCCTTCTG |
|  | universal AP | CTCAACTGGTGTCGTGGAGTC |
| U6 | SP | GCTTCGGCAGCACATATACTAAAAT |
|  | AP | CGCTTCACGAATTTGCGTGTCAT |

**Notes:** SP: sense primer, AP: anti-sense primer, RT stem-loop: primer for miRNA reverse transcript, Universal AP: universal anti-sense primer for miRNA qPCR.
