# Supplementary material for: Discovery of MicroRNAs Associated with Myogenesis by Deep Sequencing of Serial Developmental Skeletal Muscles in Pigs
Source: PLoS One. 2012 Dec 21;7(12):e52123. doi: 10.1371/journal.pone.0052123 (PMC3528764; doi:10.1371/journal.pone.0052123)
Supplement: Table S6 — Primers for luciferase reporter construction. (DOC) [file pone.0052123.s006.doc]

**Table S6 Primers for luciferase reporter construction**

| 3’UTR | primer | sequence (5'→3') |
| --- | --- | --- |
|
| BMP2 | SP | CTCGAGGACGTTGGTCAACTCCGTTAACT |
|  | AP | GCGGCCGCGTTCTCATGGACTGGGTAACCAC |
| MAPK1 | SP | CTCGAGGCTCAGACGTCGGTGTTCTTCC |
|  | AP | GCGGCCGCCATACTGCCACAGGCCACCAG |

**Notes:** SP: sense primer, AP: anti-sense primer.
